# Free Radical Scavenging and Antioxidant Activities of Silymarin Components

**DOI:** 10.3390/antiox2040398

**Published:** 2013-12-10

**Authors:** Kevin P. Anthony, Mahmoud A. Saleh

**Affiliations:** Department of Chemistry, Texas Southern University, Houston, TX 77004, USA; E-Mail: AnthonyKP@TSU.EDU

**Keywords:** *Silybum marianum*, milk thistle, food supplements, hepatoprotection

## Abstract

Silymarin is an over the counter food supplement that is sold as a liver enhancement and liver protection preparation. It is a major constituent of the seeds of *Silybum marianum* which is composed of a mixture of seven major components and several minor compounds. The seven major components: taxifolin, silychristin, silydianin, silybin A, silybin B, iso-silybin A and iso-silybin B were isolated and purified from the crude mixture of silymarin using preparative high performance liquid chromatography to determine which were the most effective for liver protection. Free radical scavenging, hydroxyl radical antioxidant capacity, oxygen radical antioxidant capacity, trolox-equivalent antioxidant capacity and total antioxidant capacity antioxidant activities were determined for each of the individual purified components as well as the crude silymarin mixture. Taxifolin was the most effective component for scavenging free radicals in the DPPH assay with an EC_50_ of 32 µM far more effective than all other components which showed EC_50_ ranging from 115 to 855 µM. Taxifolin was also found to be the most effective antioxidant in the oxygen radical antioxidant capacity (ORAC) assay with a trolox equivalent of 2.43 and the second most effective in the hydroxyl radical antioxidant capacity (HORAC) assay with a gallic acid equivalent of 0.57. Other antioxidants assays did not show significant differences between samples.

## 1. Introduction

Oxygen free radicals and other reactive oxygen species (ROS) such as superoxide anion radical O_2_**^•−^**, hydrogen peroxide (H_2_O_2_), alkoxyl (RO**·**), peroxyl (ROO**·**), hydroxyl radical (OH**·**), and hypochlorous acid (HOCl**·**), as well as reactive nitrogen species (RNS) such as nitric oxide (NO**·**) and peroxynitrite, are known to damage living tissues and cellular components. In biological systems this process is called oxidative stress or oxidative damage and has become a significant topic in the field of environmental toxicology [1,2]. Many environmental pollutants are shown to initiate oxidative damage, for example heavy metals, polycyclic aromatic hydrocarbons, pesticides, polychlorinated biphenyls, dioxins, and other xenobiotics [3,4]. Free radical reactions and the production of toxic ROS/RNS are known to be responsible for a variety of adverse health effects and diseases [5,6].

Liver cells possess a number of compensatory mechanisms to deal with ROS and its effects. Among these, are the induction of antioxidant proteins such as superoxide dismutase (SOD), catalase, and glutathione peroxidase (GSHPx). Enzymatic antioxidant systems [Cu–Zn, Mn–SOD, catalase, GSHPx, and GSH reductase (GR)] function by direct or sequential removal of ROS, thereby terminating their activities. An imbalance between the oxidative forces and antioxidant defense systems causes oxidative injury, which has been implicated in various diseases, such as atherosclerosis, diabetes, cancer, liver cirrhosis, *etc.* [5,6]. ROS are continuously generated in physiological conditions and effectively eliminated by several intracellular and extracellular antioxidant systems. Free radical reaction is an important pathway in a wide range of unrelated biological systems. Among many ways of chemical-induced injury, the critical class of reaction is production of free radical intermediates which trigger a network of multifarious disturbances. Most of the hepatotoxic chemicals damage liver cells mainly by inducing lipid peroxidation and other oxidative damages [7]. Liver possesses a unique metabolism and plays a pivotal role in the removal of substances from the portal circulation which it is susceptible to toxicity of drugs, xenobiotics, and oxidative stress [8]. The two distinct pathways in liver metabolism occur via cytochrome P450 and GSH-peroxidase. The current treatment for hepatotoxicity includes drugs which influence the P450 enzyme mechanism either by inhibiting (amiodarone, cimetidine, ciprofloxacin, *etc.*) or inducing (rifampicin, carbamazepine, phenobarbital, phenytoin) the metabolic activity of enzymes. Recently, much attention has been focused on investigating the hepatoprotective function of naturally occurring compounds and their mechanisms of action.

Silymarin is the major bioactive constituent of the milk thistle seed extract of the medicinal plant *Silybum marianum* of the family Asteraceae. Silymarin has been widely used as a therapeutic agent for a variety of acute and chronic liver diseases [9,10]. It has been used for centuries for the protection of the liver from toxic substances, treating liver damage, therapy of hepatitis and cirrhosis [11,12,13,14]. In addition to its antioxidant properties, it has been reported to have high anti-tumor promoting activity [15] and has been linked to the prevention of skin carcinogenesis [16]. Recent studies have also reported that silymarin is an effective antiviral treatment for hepatitis C virus (HCV) [17]. However, little is known about how it works [18]. Silymarin is a mixture of seven major components: taxifolin, silychristin, silydianin, silybin A, silybin B, isosilybin A and isosilybin B [19,20].

In the current study, we examined the seven main components of silymarin and determined their individual ability to quench reactive oxygen radicals and to evaluate their antioxidant activities.

## 2. Experimental Section

### 2.1. Chemicals and Reagents

All solvents used for HPLC and MS analyses were of hplc/ms chromatographic grade. Formic acid and dimethyl sulfoxide (DMSO) were purchased from VWR International Co. (Sugar Land, TX, USA). Technical silymarin (>96% pure) taxifolin, silychristin, silydianin, silibinin, iso-silybin A and B and 2,2-diphenyl-1-picrylhydrazyl (DPPH) were purchased from Sigma Aldrich Inc., Atlanta GA. Silybin A and B were isolated from silibinin (1:1 mixture of silybin A and B) purchased from Sigma Aldrich.

### 2.2. Isolation and Purification of the Individual Silymarin Components

All of the individual components of silymarin were isolated from silymarin (Sigma Products) using high resolution preparative HPLC Dionex Summit systems (Sunnyvale, CA, USA) equipped with P680 HPLC pump, solvent delivery module, auto sampler, automatic sampler injector, (117-well capacity), controller module, column oven, photodiode array detector (PDA), with data collected and analyzed using Star Chromeleon chromatography managing system software (version 6.80). Phenomenex (Luna C_18_ AXIAP 5 micron) column of 250 mm in length and 21.2 mm in diameter was used for the separation. Fractionation was carried out with a isocratic mobile phase of methanol: 0.1% formic acid in water (60:40, by volume) at a flow rate of 20 mL/min. Column effluent were split to 1:100 using an QuickSplitTM flow splitter (Analytical Scientific Instruments, Richmond, CA 94806, USA), 1% of the column effluent was directed to the detector while 99% of the effluent went to the collector. Peaks were detected at 288 nm. Aliquots of silymarin (Sigma) dissolved in DMSO were repeatedly injected in the HPLC using 300 µL per injection containing 100 mg of crude product. To achieve the highest purity, each of the individual peaks was collected manually at its half highest peak front to half height of peak ends. Each individual peak collected was examined by analytical LCMS to ensure purity of 95% or higher. Purity of individual components was evaluated using mass, ultraviolet spectral data, retention times, and co-chromatography with Sigma standard chemical. Taxifolin (50 mg), silychristin (25 mg), silydianin (20 mg), silybin A (160 mg), silybin B (250 mg), isosilybin A (15 mg), and isosilybin B (10 mg) were obtained.

### 2.3. Free Radical-Scavenging Activity: DPPH Test

Free radical-scavenging activity of each silymarin components was carried out using the DPPH scavenging method [21]. The test was carried out using Perkin Elmer Victor 4X micro plate reader performed in a 96 well plate using a total volume of 200 µL methanol containing 0.004 µg DPPH and samples aliquots at a series of concentrations of 1, 0.5, 0.25, and 0.125 µg/mL. The test was repeated at all concentration of each sample in triplicate. DPPH solutions at the same concentration without the tested samples were used as control. Each sample, as well as each control was analyzed in triplicates. After filling the well plates, they were loaded into the plate reader, incubated at 25° and read every 5 min for 30 min at 520 nm. The free radical scavenging activity of each solution was then calculated as percent inhibition according to the following equation:

% Inhibition = 100 × (*A*_blank_ − *A*_sample_)/*A*_blank_ (1)

where *A*_sample_ is the absorbance of the sample and *A*_blank_ is the absorbance of the blank. Inhibition % was plotted against concentration and the EC_50_ was calculated graphically.

### 2.4. Hydroxyl Radical Antioxidant Capacity (HORAC)

The HORAC activity assay is based on the oxidation of a fluorescent probe (fluorescein) by hydroxyl radical via hydrogen atom transfer (HAT) process [22]. Hydroxyl radicals were produced by hydroxyl radical initiator (H_2_O_2_) and fenton reagent, which quenches the fluorescent probe over time. Antioxidants present in the assay work to block the radical hydroxyl oxidation of the fluorescent probe until the antioxidant activity in the sample is depleted. The sample antioxidant capacity correlates to the fluorescence decay curve and is used to quantify the total hydroxyl radical antioxidant activity in a sample and is compared to a gallic acid antioxidant standard curve. The assay was carried out using commercial assay kit (OxiSelect™ Hydroxyl Radical Antioxidant Capacity (HORAC) Activity Assay/STA-346, Cell Biolabs, Inc., San Diego, CA, USA). The test was carried out using samples aliquots at concentrations of 500 and 700 µM. Each sample, as well as each control was analyzed in triplicates and the results are reported as the average of the three measurements with standard deviations.

### 2.5. Oxygen Radical Antioxidant Capacity (ORAC)

The ORAC Activity Assay is based on the oxidation of fluorescein as a fluorescent probe by peroxyl radicals by way of a hydrogen atom transfer (HAT) process. Peroxyl radicals are produced by a free radical initiator (2,2′-Azobis (2-methylpropionamidine) hydrochloride (AAPH)) which quenches the fluorescent probe over time. Antioxidants present in the assay work to block the peroxyl radical oxidation of the fluorescent probe until the antioxidant activity in the sample is depleted. The remaining peroxyl radicals destroy the fluorescence of the fluorescent probe. The sample antioxidant capacity correlates to the fluorescence decay curve, which is used to quantify the total peroxyl radical antioxidant activity in a sample and is compared to an antioxidant standard curve of the water soluble vitamin E analog Trolox. The assay was carried out using commercial assay kit (OxiSelect™ Hydrogen Peroxide Assay Kit (Colorimetric) Activity Assay/STA-343, Cell Biolabs, Inc., San Diego, CA, USA). The hydrophobic protocol of the kit was performed using samples at concentrations 0, 2.5, 5, 10, 20, 30, 40 and 50 uM. Results were acquired after one hour of average reading at excitation wave length of 485 nm and emission at 520 nm.

### 2.6. Total Antioxidant Capacity (TAC)

The TAC Assay is based on the reduction of copper (II) to copper (I) by antioxidants such as uric acid as described by Bakir *et al*. [23]. Upon reduction, the copper (I) ion further reacts with a coupling chromogenic reagent that produces a color with a maximum absorbance at 490 nm. The net absorbance values of antioxidants are compared with a known uric acid standard curve. Absorbance values are proportional to the sample’s total reductive capacity. Results are expressed as μM Copper reducing equivalents or mM uric acid equivalents. A fresh Uric Acid standard was prepared by weighing out the Uric Acid powder for a 10 mg/mL solution in 1 N NaOH. This 10 mg/mL is equivalent to a concentration of 60 mM. The 60 mM Uric Acid solution was used to prepare a 2 mM solution of Uric Acid (e.g., add 100 μL of the 60 mM Uric Acid standard to 2.900 mL of deionized water). Each sample was prepared using the 2 mM concentration. Five different concentrations were used for the sample analysis, 0.5, 0.25, 0.625 and 0.03125 mM. 20 uL of 2 mM sample stock was added to 180 uL of the 1× Reaction buffer and mixed. An initial reading was taken at 490 nm. Then 50 uL of the 1× Copper Ion reagent was added and incubated for 5 min on an orbital shaker. Then 50 uL of the Stop solution was added to terminate the reaction and the plate was read again at 490 nm.

### 2.7. Trolox-Equivalent Antioxidant Capacity Assay

Trolox-equivalent antioxidant capacity (TEAC) of the seven individual components was carried out using the procedure described in the antioxidant assay kit (item No. 709001 from Cayman Chemical Company1180 E. Ellsworth Rd. Ann Arbor, MI, USA). Samples were prepared at seven concentrations of 0, 0.24, 0.48, 0.72, 0.96, 1.2 and 2.4 µM using a 96 well plate. 10 µL of this preparation was removed and added to 10 µL of metmyoglobin, 150 µL of chromogen and 40 µL of hydrogen peroxide mixture for a total of 210 µL in each well. The plate was covered and placed on a shaker for five min and read at 750 nm using a Perkin Elmer Victor X4 2030 Multilabel Reader (710 Bridgeport Avenue Shelton, CT, USA). The absorbance was plotted as a function of the final trolox concentration (mM) according to the assay protocol.

Antioxidant (mM) = Sample absorbance − (*y* − intercept)/Slope × Dilution (2)

Each sample, as well as each control was analyzed in triplicate and the results are reported as the average of the three measurements with standard deviations.

## 3. Results and Discussion

**Figure 1:**
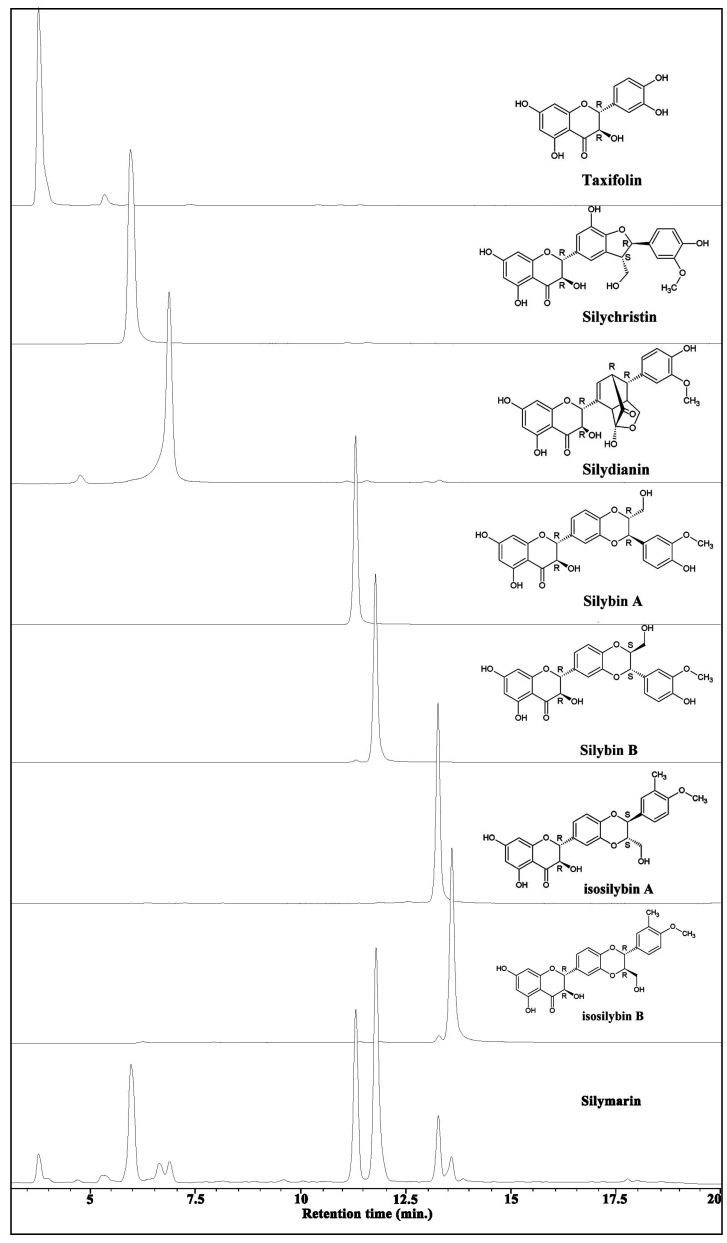
Total ion chromatograms of silymarin and its purified components and their chemical structures.

Total ion chromatogram of silymarin and all of the seven individual components with their chemical structures are shown in Figure 1. All purified compounds were having at least 95% purity as shown by analytical HPLC both in the UV scan as well as with HPLCMS.

**Figure 2:**
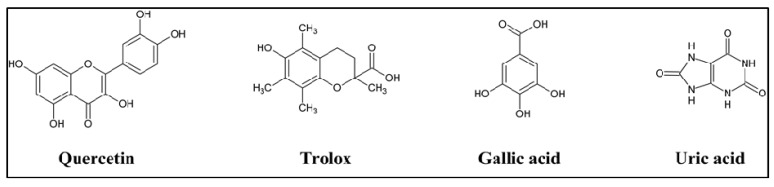
Chemical structure of standard compounds used as positive controls for antioxidant activity.

DPPH was used to evaluate the ability of the silymarin individual components compared to the crude silymarin mixture to act as free radical scavengers by determining their EC_50_ (lower values indicate higher radical scavenging power). Taxifolin was found to be exceptionally much more active as radical scavenger compared to the silymarin mixture and other silymarin components with an EC_50_ of 32 µM (Table 1). Taxifolin is the only component of silymarin that is not of the flavolignanes group and is related to the quercetin (dehydrotaxifolin, Figure 2) which is known to have powerful radical scavenging activity [24]. Isosilybin A and B were the least active of the seven components for scavenging free radicals with an EC_50_ of 855 and 813 µM respectively. Silychristin and silydianin were moderately active but more active than other tested isomers.

Total hydroxyl radical antioxidant activity of silymarin and its components were measured and compared to standard reference compound gallic acid (Figure 2). The results are reported as equivalents to gallic acid, given the activity of gallic acid as 1. All tested samples were less effective than gallic acid for destroying the hydroxyl radicals (Table 1). However, taxifolin, silychristin and silydianin were more effective than the crude mixture of silymarin or all other components.

**Table 1:** Radical scavenging and antioxidant activities of silymarin and its individual components.

Chemicals	Radical scavenging	Antioxidant activity			
	DPPH EC_50_, µM	HORAC gallic acid equivalent	ORAC peroxyl radical trolox equivalent	TAC * uric acid equivalent	ABTS trolox equivalent
Taxifolin	32 ± 1.0	0.57 ± 0.03	2.43	1.69 ± 0.05 (3699)	0.75 ± 0.03
Silychristin	130 ± 3.9	0.66 ± 0.08	0.65	1.88 ± 0.02 (4116)	0.61 ± 0.06
Silydianin	115 ± 3.5	0.46 ± 0.01	0.59	1.64 ± 0.01 (3599)	0.85 ± 0.08
Silybin A	311 ± 9.3	0.42 ± 0.04	0.33	2.08 ± 0.20 (4560)	0.82 ± 0.04
Silybin B	344 ± 10	0.41 ± 0.02	0.31	2.14 ± 0.21 (4673)	0.79 ± 0.02
iso-Silybin A	855 ± 26	0.38 ± 0.03	0.25	0.90 ± 0.07 (1978)	0.98 ± 0.06
iso-Silybin B	813 ± 24	0.38 ± 0.02	Not Tested	1.12 ± 0.02 (2450)	1.05 ± 0.21
Silymarin	280 ± 8.4	0.42 ± 0.02	1.18	1.43 ± 0.01 (3131)	0.97 ± 0.12

* Values in parenthesis show Cu^II^ to Cu^I^ reduction equivalent.

Peroxyl radical antioxidant activity were determined in all of the individual components of silymarin and silymarin mixture, expressed as equivalent to trolox (Figure 2), which is a water soluble vitamin E analog and known to have powerful oxygen radical inhibition [25]. All components showed lower activity than silymarin. However, taxifolin was much a more effective antioxidant than the crude silymarin mixture, showing as much as twice the effectiveness of whole silymarin.

The TAC Assay is based on the reduction of copper (II) to copper (I) by uric acid and the results are shown as reducing equivalents to uric acid and as μM copper reducing equivalents (Table 1). All individual purified components were slightly more effective than crude silymarin except iso-silybin A and B, which were less effective.

Trolox equivalents were very similar among all of the different silymarin individual components as well as the crude mixture of silymarin suggesting that this test may not be as sensitive as other tests described above for evaluating antioxidant activity for the silymarin products.

From all of the results described above, it is clear that silymarin and some of its active constituent could have potential beneficial effects as antioxidants in agreement with results reported by other investigators [26,27,28]. However, their work was only carried out on the crude silymarin mixture. Ramasamy and Agarwal [26] showed that silymarin may act as a multitargeted therapy for cancer by acting as an antioxidant. Svobodova and others [27] showed that silymarin and silibinin exert anti-oxidant activity and support redox homeostasis in several *in vitro* and *in vivo* models.

## 4. Conclusions

**Figure 3:**
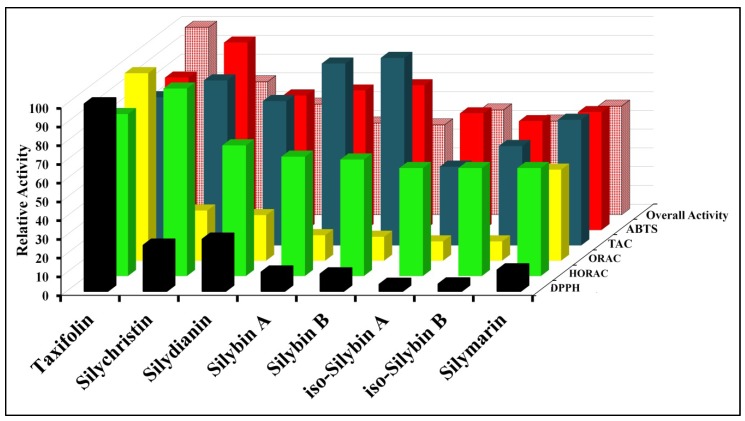
Overall antioxidant activity of silymarin and its individual components.

Based on the above results, it can be concluded that taxifolin is the most effective component of silymarin, as can be seen mainly from Free Radical-Scavenging and Hydroxyl Radical Antioxidant Capacity assays (Figure 3). Taxifolin is the only isomer of silymarin that is not of the flavolignanes group and is related to the quercetin (dehydrotaxifolin), known to have powerful antioxidant activity [24]. Based on our previous work [20], taxifolin is only 3.5% of the total silymarin mixture but it is the major contributor to the antioxidant activity of silymarin. Silybin A and silybin B are 17% and 31% respectively making up approximately 50% of the silymarin mixture, but have much lower antioxidant activity. In addition, taxifolin was also found to have no cytotoxicity [10], therefore, selective breading of *Silybum marianam* to increase taxifolin contents may enhance the antioxidant activity of silymarin and improve its effectiveness as an over the counter liver protection food supplement.
